# Sustained efficacy following resolution of frequent heartburn with an over-the-counter regimen of esomeprazole 20 mg or placebo for 14 days: two randomized trials

**DOI:** 10.1186/s12876-018-0790-2

**Published:** 2018-05-22

**Authors:** David A. Peura, Anne Le Moigne, Heather Wassel, Charles Pollack

**Affiliations:** 1 University of Virginia, School of Medicine, Charlottesville, VA 22908 USA; 2 Clinical Excellence and Biometrics, Pfizer Consumer Healthcare, Madison, NJ 07940 USA; 3 Pfizer Consumer Healthcare, Madison, NJ USA; 4 Former Senior Director, Global R&D, Pfizer Consumer Healthcare, Madison, NJ 07940 USA; 5 Department of Medicine, Division of Gastroenterology and Hepatology, University of Virginia, School of Medicine, 1215 Lee Street, Charlottesville, VA 22908-0708 USA

**Keywords:** Esomeprazole, Proton pump inhibitor, Heartburn, Gastric acid/secretion

## Abstract

**Background:**

A two-week course of therapy with an over-the-counter proton-pump inhibitor (PPI) is recommended for frequent heartburn. Limited research has been conducted on the sustained efficacy of short-term PPI therapy after treatment cessation. Esomeprazole 20 mg was evaluated in the seven-day follow-up period after the two-week treatment period using pooled data from two identical randomized, double-blind, placebo-controlled studies.

**Methods:**

Adults without confirmed diagnoses of gastroesophageal reflux disease experiencing heartburn at least two days/week in the past four weeks were eligible. Subjects received treatment with esomeprazole 20 mg or placebo once daily for 14 days. Heartburn episodes were documented using daily diaries. Missing data during the two-week treatment period were assumed to be days with heartburn. The proportion of subjects with heartburn resolution while on treatment and during the seven days of follow-up was assessed. Predictors of resolution during this post-treatment period were evaluated using a stepwise logistic regression model.

**Results:**

All subjects in the pooled analysis set who reported diary data for at least three follow-up days were analyzed (*N* = 584). This cut-off was used to ensure that sufficient data were collected for these analyses. Greater run-in heartburn frequency was a significant negative predictor of heartburn resolution during follow-up (*P* < 0.001). Among the on-treatment efficacy variables, the best predictor of resolution during follow-up was resolution during the last seven days of treatment (odds ratio: 3.81 [95% confidence interval: 2.40, 6.05; *P* < 0.0001]).

**Conclusions:**

Lower baseline heartburn frequency and heartburn resolution during the last seven days of treatment were associated with a greater likelihood of heartburn resolution during the seven-day follow-up.

**Trial registration:**

Registered at ClinicalTrials.gov June 11, 2011: NCT01370525; NCT01370538.

## Background

Frequent heartburn and regurgitation occur commonly in the global population, with incidence rates ranging from approximately 10% to 30% in Western countries [1]. Proton-pump inhibitors (PPIs) are highly effective for treating these symptoms [2] and have become available worldwide without a prescription for self-treating frequent heartburn [3, 4]. Esomeprazole is a PPI that has recently become available over-the-counter (OTC) for treating frequent heartburn at a dose of 20 mg once daily for 14 days [5]. The efficacy of esomeprazole in the OTC setting has been demonstrated in two randomized, double-blind, placebo-controlled, phase 3 clinical trials in adults with frequent heartburn [6].

The World Gastroenterology Organization’s guidelines for treating frequent heartburn (two or more days/week) recommend a two-week course of treatment with an OTC PPI along with lifestyle and dietary modifications [3]. In the US, OTC esomeprazole 20 mg is approved for 14 days of treatment for frequent heartburn, a treatment course that can be repeated once every four months; however, if symptoms persist or recur within this time frame the individual should consult a physician [3, 5]. The effects of discontinuing PPI therapy have been studied primarily in the context of rebound acid hypersecretion after long-term use and the development of effective discontinuation strategies to reduce unnecessary use of PPIs [7, 8]. However, analyses assessing the degree to which the symptomatic response is sustained after discontinuation of a short-term course of OTC PPIs are limited. Two studies conducted with an OTC PPI failed to show continued benefit during the week after the completion of active treatment [9]. Determining whether the level of heartburn resolution while on treatment has an impact on the duration of response following treatment cessation is therefore important to clinical practice.

The current post hoc analyses were conducted with data collected during two phase 3 clinical trials that assessed a 14-day treatment regimen with esomeprazole 20 mg or placebo once daily in subjects experiencing frequent heartburn who are likely to self-treat these symptoms [6]. The primary objective of these analyses was to evaluate the impact of reaching resolution of heartburn at different time points throughout the treatment period on the durability of response during a one-week follow-up period at the end of active treatment. Additionally, a secondary objective was to analyze subject-level data to determine clinical factors that may be related to experiencing a sustained treatment response.

## Methods

NEXT-1 and NEXT-2 were two identical phase 3, randomized, double-blind, placebo-controlled studies designed to determine the efficacy of a 14-day regimen of esomeprazole 20 mg for treating frequent heartburn in subjects who are likely to self-treat with OTC medications without consulting a healthcare provider. Data from these studies, which were registered at ClinicalTrials.gov under identifiers NCT01370525 and NCT01370538, were pooled for the current analyses. The methodology for conducting these studies has been published in detail previously [6] and is summarized briefly here.

### Study design

Both studies enrolled otherwise healthy male and female subjects aged ≥18 years who had experienced frequent heartburn on two or more days per week over the prior four weeks. Exclusion criteria included confirmed diagnosis of gastroesophageal reflux disease (GERD) or any history of erosive esophagitis, current use of prescription medications for GERD, or a need for continuous treatment with prescription histamine H_2_ receptor antagonists (H_2_RAs), PPIs, gastric prokinetic drugs, or antacids throughout the study for any indication. Subjects who required more than one 14-day course of PPI therapy over the past four months and three or more 14-day courses of PPI therapy during the past year were excluded.

Informed consent was obtained from all individual participants included in these studies, which were conducted in accordance with the Declaration of Helsinki. The informed consent form and study protocol for each study were approved by Alpha Independent Review Board (San Clemente, California, USA) [6].

Following heartburn medication washout (one or more days for antacids and seven or more days for H_2_RAs and/or PPIs), eligible subjects were enrolled in a seven-day placebo run-in period. After completing the run-in period, subjects who continued to meet the inclusion criteria and were compliant with reporting heartburn symptoms were randomly assigned to 14 days of double-blind treatment with either esomeprazole 20 mg or placebo administered once daily. At the end of the 14-day active treatment period, all subjects subsequently entered a one-week single-blind placebo follow-up period. Because the investigators were aware of this aspect of the study design, only subjects remained blinded to the treatment they were receiving during the follow-up period. Although unblinding the investigators generally carries the risk of introducing bias, the subjects were responsible for recording their symptoms, so sustaining the blind for the subjects would not be expected to have an impact on the reporting of their symptoms.

### Treatments

Eligible subjects were randomly assigned to receive 14 days of double-blind treatment with esomeprazole 20 mg capsules (administered as esomeprazole magnesium trihydrate 22.3 mg) or matching placebo. To reflect the instructions for using OTC PPIs, subjects were instructed to take study drugs once daily before eating their morning meal. Subjects were permitted to use antacid tablets (Gelusil® tablets; WellSpring Pharmaceutical Corporation, Sarasota, Florida, USA) as a rescue medication for managing breakthrough symptoms; however, the use of other prescription or OTC heartburn treatments was prohibited during the study period.

### Outcome measures of current analyses

The primary efficacy endpoint for both studies was the percentage of heartburn-free 24-h days during the 14 days of treatment as measured by daily self-assessment diaries that utilized an interactive voice response system. Subjects were asked to rate their overall heartburn severity over the past 24 h using the following scale: 0 = none; 1 = mild; 2 = moderate; 3 = severe. The primary results, which demonstrate significant improvements for esomeprazole 20 mg over placebo on all primary and secondary endpoints, have been previously published [6].

The primary efficacy outcome of interest for the current analyses was resolution of heartburn during the follow-up period, which was defined as one or no days of heartburn. Secondary endpoints for these analyses included heartburn resolution and the number of heartburn-free days during weeks 1 and 2 individually, over the entire two-week treatment period, and during the last seven days of treatment, which included the last seven consecutive days when the subjects received randomized study medication. Resolution was defined as one or no days (week 1 and 2, and last seven days of treatment) or two or fewer (two-week treatment period) days of heartburn during the period of interest. These outcomes were all planned secondary endpoints in the two phase 3 trials.

### Statistical analyses

The analyses presented in this report are based on pooled data from the full analysis set from NEXT-1 and NEXT-2. The full analysis set consisted of all subjects who received at least one dose of randomized treatment and had a baseline and at least one post-baseline efficacy measurement recorded. Additionally, subjects in the pooled full analysis set who had reported diary data for three or more days during the seven-day follow-up period were included in these analyses. Because these analyses focused on symptom response during the follow-up period, this cut-off was used to ensure that adequate data were collected during this period to evaluate heartburn resolution without excluding too many subjects.

Stepwise logistic regression models were developed to identify variables during the run-in and on-treatment periods that were the best predictors of resolution in the follow-up period, regardless of treatment group assignment. Variables included in the first model were the number of days with heartburn during the run-in period and heartburn resolution during week 1, week 2, the entire two-week treatment period, and the last seven days of treatment. Variables included in the second model were the number of days with heartburn during the run-in period and number of days without heartburn during week 1, week 2, the entire two-week treatment period, and the last seven days of treatment. A mixed-effect model was also used to determine which variables were good predictors of the number of days with heartburn during the follow-up period. Variables included in this model were the number of days without heartburn during week 1, week 2, the entire two-week treatment period, and the last seven days of treatment. All of the mixed models included the number of days with heartburn during the run-in period as a covariate. The proportion of subjects with heartburn resolution and the number of heartburn-free days during each treatment period investigated are reported with descriptive statistics.

## Results

A total of 584 subjects had complete diary data for at least three days during the placebo follow-up period, representing 89.7% of the total full analysis set population enrolled in the two studies (*n* = 651). Baseline demographics for this population are presented in Table 1 and are similar to the complete study population for the two trials [6]. Subjects in this subgroup were primarily female (56–57%) and 43–45 years of age and experienced a mean of 5.7 days of heartburn during the run-in period. In the total study population, 55–58% of subjects were female, and their mean age (43–44 years) and number of run-in heartburn days (5.7–5.8) were generally consistent with the subgroup used for these analyses.

**Table 1:** Baseline demographics

Characteristic	Esomeprazole 20 mg (*n* = 294)	Placebo (*n* = 290)
Age, years		
Mean (SD)	43.1 (13.0)	44.9 (12.6)
Female, n (%)	166 (56.5)	162 (55.9)
Race, n (%)		
White	186 (63.3)	200 (69.0)
Black/African American	100 (34.0)	87 (30.0)
Asian American Indian/Alaskan native	0 (0) 3 (1.0)	1 (0.3) 0 (0)
Native Hawaiian/Pacific Islander	1 (0.3)	0 (0)
Other	4 (1.4)	2 (0.7)
Run-in Heartburn Frequency, days		
Mean (SD)	5.7 (1.8)	5.7 (1.8)

*SD* standard deviation

### Resolution and number of heartburn-free days

**Fig. 1:**
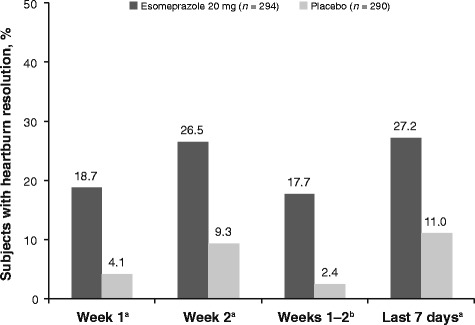
Percentage of subjects with heartburn resolution at selected time points during treatment. Days with missing data were assumed to be days with heartburn. ^a^One or no days with heartburn. ^b^Two or fewer days with heartburn

**Fig. 2:**
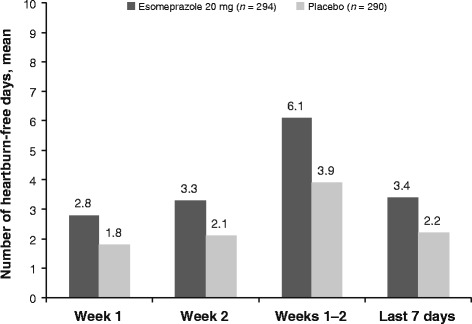
Mean number of heartburn-free days at selected time points during treatment. Days with missing data were assumed to be days with heartburn

During each on-treatment time interval that was analyzed, subjects treated with esomeprazole 20 mg had higher rates of resolution (defined as one or no or two day(s) with heartburn) versus those treated with placebo (Fig. 1). During the last seven days of treatment, 27.2% of subjects treated with esomeprazole 20 mg had resolution of heartburn, compared with 11.0% in the placebo group. Similarly, as shown in Fig. 2, the mean number of heartburn-free days was higher for subjects in the esomeprazole 20 mg group versus the placebo group during each period. During the last seven days of treatment the mean number of heartburn-free days was 3.4 and 2.2 in the esomeprazole 20 mg and placebo groups, respectively. The mean number of heartburn-free days during the entire two-week treatment period for esomeprazole 20 mg and placebo was 6.1 days and 3.9 days, respectively.

### Predictors of heartburn resolution during follow-up period

**Fig. 3:**
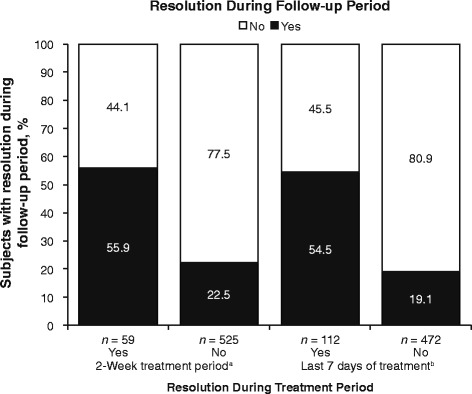
Heartburn resolution status at follow-up by status during the on-treatment period. ^a^Two or fewer days with heartburn. ^b^One or no days with heartburn

**Fig. 4:**
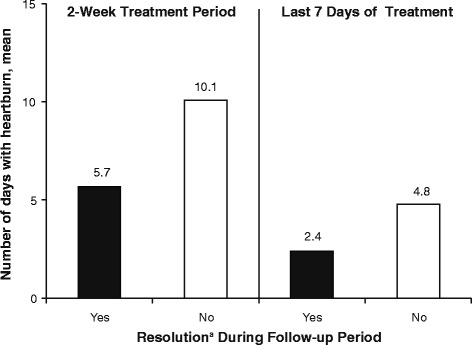
Number of days with heartburn during the on-treatment period by resolution status at follow-up. ^a^One or no days with heartburn

Of the subjects who achieved heartburn resolution during the last seven days of treatment, 54.5% maintained resolution in the follow-up period, compared with 19.1% of those who had not achieved resolution during the last seven days of treatment (Fig. 3). These same subjects had a mean of 5.7 days with heartburn during treatment, compared with 10.1 days for subjects not achieving heartburn resolution during the follow-up period (Fig. 4).

The stepwise logistic regression model identified several key predictors of heartburn resolution during the placebo follow-up period, independent of treatment randomization to esomeprazole 20 mg or placebo. This model demonstrated that a greater frequency of run-in heartburn was a significant negative predictor of heartburn resolution during the follow-up period, with an adjusted odds ratio (OR) of 0.68 (95% confidence interval [CI]: 0.61–0.77) for each additional 1-day increase in heartburn frequency during the run-in phase (*P* < 0.001 in both logistic regression models; Table 2). Among the on-treatment efficacy variables included in the stepwise logistic regression model focusing on resolution parameters, the best predictor of heartburn resolution during the follow-up period was heartburn resolution (one or no days of heartburn) during the last seven days of treatment, with an OR of 3.81 (95% CI: 2.40–6.05; *P* < 0.0001; Table 2). For the stepwise logistic regression model that was conducted to identify predictors of heartburn resolution during the follow-up period with independent variables, focusing on the number of heartburn-free days in each treatment period (i.e., week 1, week 2, two weeks of treatment, and the last seven days of treatment), the best predictor of heartburn resolution was the number of days without heartburn during the full two-week treatment period. The OR for this endpoint was 1.24 (95% CI: 1.17–1.30; *P <* 0.0001), indicating that while holding the number of days with heartburn during run-in at a fixed value, every 1-day increase in heartburn-free days during the two-week treatment period increased the odds of heartburn resolution during the follow-up period by 24% (Table 2).

**Table 2:** Predictors of heartburn resolution during the follow-up period

Independent Variables Selected for Final Models		
Resolution		Adjusted Odds Ratio (95% Wald CI)
No. of days with HB during run-in period	For every 1-day increase in run-in HB frequency	0.68 (0.61–0.77)
Resolution during the last seven days	Yes vs. no	3.81 (2.40–6.05)
No. of HB-Free Days		Adjusted Odds Ratio (95% Wald CI)
No. of days with HB during run-in period	For every 1-day increase in run-in HB frequency	0.79 (0.69–0.89)
No. of HB-free days during two weeks of treatment	For every 1-day increase in HB-free days	1.24 (1.17–1.30)

*CI* confidence interval, *HB* heartburn

In a separate mixed model analysis that was conducted to identify predictors of the number of days with heartburn during the follow-up period, the best model included the number of days without heartburn during the entire two-week treatment period. For every additional day without heartburn during the entire two-week treatment period there was a reduction in the number of days with heartburn during the seven-day follow-up period of 0.22 days (95% CI: 0.26–0.18; *P* < 0.0001). These results indicate that, for example, holding the number of days with heartburn during the run-in as a constant value, if an individual experienced 10 heartburn-free days while on treatment it is estimated that they would have 2.2 fewer days of heartburn during follow-up than if they had experienced zero heartburn-free days while on treatment. The number of days with heartburn during the seven-day run-in period was also predictive of the number of days with heartburn during the follow-up period. For each additional day of heartburn during the run-in period the number of days with heartburn during the follow-up period increased by 0.27 days (95% CI: 0.18–0.35; *P* < 0.0001).

## Discussion

The current analyses demonstrate that a greater frequency of heartburn prior to initiating treatment with esomeprazole 20 mg was associated with a decreased likelihood of sustained symptom resolution during the seven days after the cessation of treatment. In the publication describing the secondary endpoints of these studies, which also included a post hoc, pooled analysis of heartburn-free days during the follow-up period, the between-group differences on this outcome favored esomeprazole 20 mg significantly over placebo (difference: 5.7 [95% CI: 1.0–10.5; *P* = 0.018) [10]. Additionally, the number of heartburn-free days remained significantly higher *(P* < 0.001) during the post-treatment period than the run-in phase, indicating that there was no symptomatic rebound at the end of the active treatment phase. To our knowledge, no studies that examined the degree to which treatment response is sustained following the discontinuation of short-term PPI therapy have demonstrated a significant effect after treatment has ended. During two similarly designed studies that were conducted with lansoprazole 15 mg for 14 days for treating frequent heartburn, no significant differences between active treatment and placebo were observed in the percentage of heartburn-free days (44 to 47%) during the 7-day placebo follow-up periods [9].

A prior analysis of two different esomeprazole trials that were conducted to assess the effect of treatment on reflux-related sleep disturbances found that a higher frequency of sleep disturbances during the run-in period was associated with a lower rate of sleep disturbance resolution during the 14-day treatment period [11]. Additionally, an absence of daytime or nighttime heartburn during the active treatment period was associated with a greater likelihood of resolution of reflux-related sleep disturbances. Bardhan et al. demonstrated that subjects receiving an initial two weeks of treatment with ranitidine or omeprazole followed by an additional two- to four-week course of on-demand treatment were able to remain off treatment for a median of 142 days, with only 22% of those receiving omeprazole being transferred to maintenance therapy [12]. Additionally, following four weeks of treatment for non-erosive GERD with rabeprazole 20 mg, continued on-demand treatment during the six months of follow-up was low, with an average intake of rabeprazole of 0.2–0.3 tablets/day [13]. These results suggest a durability of treatment effects following the resolution or significant reduction of reflux symptoms (one or no days/week) with short-term PPI treatment.

PPIs treat reflux-related symptoms by irreversibly binding to gastric proton pumps, which suppresses acid secretion, an effect that can last for as long as two days following the cessation of treatment [14]. Pharmacokinetic analyses of esomeprazole 20 mg report a half-life of approximately one hour in plasma [15], which does not provide an accurate representation of the continued pharmacodynamic effects of esomeprazole after treatment has ended. Because esomeprazole irreversibly binds to gastric proton pumps, the recurrence of symptoms at the end of active treatment is primarily dependent upon new proton pumps being synthesized [14, 16–18]. This process of restoring acid secretion following single dose administration of PPIs has been estimated to have half-times ranging from 14 to 46 h [14, 16, 18, 19].

Rebound acid hypersecretion is felt to underlie the rapid return of dyspeptic/reflux symptoms following the cessation of PPI therapy. This phenomenon has been observed primarily in healthy volunteers [20, 21], and studies conducted in individuals with heartburn have found little evidence of rebound acid hypersecretion or symptom rebound following short-term or on-demand PPI therapy [22, 23]. The potential for rebound acid hypersecretion and symptom rebound in individuals who self-diagnose and self-treat with OTC PPIs could therefore be of potential concern, but it remains to be elucidated what the level of risk is in this population. Importantly, the data presented here suggest there is no evidence of symptom rebound although rebound acid hypersecretion was not studied.

In these analyses, the most important predictors of sustained symptom control during the follow-up period were a lower incidence of heartburn frequency at baseline, heartburn resolution during the last seven days of treatment, and a greater number of days without heartburn during the two-week treatment period. In the previously described study of intermittent omeprazole treatment, which is similar to the current study, the most important predictor of sustained symptom control was response to the initial two weeks of treatment [12]. The analyses of predictors of response conducted here included subjects who received both esomeprazole and placebo, so resolution of symptoms following the treatment period in these analyses relates to symptoms during treatment, rather than to a specific treatment (esomeprazole 20 mg vs placebo). However, only a small minority of placebo-treated patients experienced heartburn resolution during the two-week treatment period, and treatment with esomeprazole was the best predictor of symptom resolution during that period [6]. Overall, we think these results can inform decision-making about the most appropriate treatment options for those with heartburn by indicating that individuals who do not achieve resolution of their heartburn during the last week of a two-week course of PPI therapy may require a change in therapy due to the increased risk for recurrences. Furthermore, because of the potential safety concerns associated with high-dose, long-term PPI therapy [3], initiating treatment for heartburn with an OTC PPI in an attempt to limit unnecessary exposure should be considered.

Although the data presented here are based on results from two well-controlled phase 3 studies, these analyses have some weaknesses that are worth noting. The data included in these analyses were collected prospectively, but the current analyses were post hoc in nature. Furthermore, while we have speculated on the mechanisms behind the continued resolution of heartburn symptoms after treatment cessation, future prospective research that explores this topic may benefit from monitoring gastric acid secretion during the post-treatment period to determine the degree to which antisecretory effects are sustained in a quantitative as well as a qualitative manner. Finally, the post-treatment follow-up period reported here extended for only seven days, so future studies should aim to evaluate symptom control during a period of time that more accurately reflects real-world use.

## Conclusions

The results of these post hoc analyses indicate that the degree of symptom resolution observed during esomeprazole therapy that is consistent with OTC use is associated with the degree of continued symptom relief following the cessation of treatment. Less-frequent pre-treatment heartburn, achieving heartburn resolution during the last seven days of treatment, and fewer days with heartburn throughout the treatment period were predictors of sustained heartburn resolution during the seven-day follow-up period that subjects entered after discontinuing 14 days of treatment with esomeprazole 20 mg. Among individuals with frequent heartburn who are likely to self-treat their symptoms without consulting a healthcare provider, esomeprazole 20 mg for 14 days in the OTC setting is associated with sustained therapeutic benefits, including after the treatment period has ended.

## Abbreviations

CI: Confidence interval
GERD: Gastroesophageal reflux disease
H_2_RA: Histamine H_2_ receptor antagonist
HB: Heartburn
OR: Odds ratio
OTC: Over-the-counter
PPI: Proton-pump inhibitor
SD: Standard deviation
